# Small Molecular Dibenzochalcogenophenes as Potent Organic Emitters

**DOI:** 10.1002/chem.202501977

**Published:** 2025-08-28

**Authors:** Marco Schmiedtchen, Lea Höfmann, Jan Balszuweit, Jens Voskuhl

**Affiliations:** ^1^ Faculty of Chemistry (Organic Chemistry) Center for Medical Biotechnology (ZMB) and Center for Nanointegration Duisburg‐Essen (CENIDE) University of Duisburg‐Essen Universitätsstraße 7 45177 Essen Germany

**Keywords:** chalcogen heterocycles, dibenzochalcogenophenes, fused‐ring systems, luminescence, photophysics

## Abstract

Dibenzochalcogenophenes (DBC) have attracted huge attention ever since the discovery that they are able to act as potent (phosphorescent) emitters more than 60 years ago. From then on, they were extensively used as versatile emissive platforms for applications in materials science and optoelectronics. In this review, we focus on the main achievements of the past 20 years, highlighting especially monomolecular small purely organic compounds with less than 10 aromatic units and only one chalcogenophene moiety. Besides this, we wish to focus on their emission properties and to evaluate general design principles to fine‐tune photophysical properties.

## Graphical abstract

This review compiles recent achievements and applications of purely organic small luminescent DBC. We present frequently employed functionalities and design strategies, accomplishing room temperature phosphorescence (RTP). In addition, we discuss the challenges for luminescent selenium and tellurium derivatives as well as the influence of commercial impurities on the photophysical properties of this compound class.

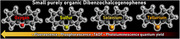

## 1 Introduction

In the manifold fields of applications for luminophores, such as bioimaging,^[^ ^1^, ^2^ ^]^ anti‐counterfeiting^[^ ^3^ ^]^ or OLEDs,^[^ ^4^ ^]^ there is a continuous trend toward the development of metal‐free emitters. Compared to their inorganic counterparts, purely organic luminophores often benefit from lower toxicity, less environmental harm, improved resource availability, and more diverse design possibilities.^[^ ^5^ ^]^ Especially the delayed emission of room‐temperature phosphorescence (RTP) is attractive for the above‐mentioned fields, for example, due to the elimination of autofluorescence background in imaging.^[^ ^2^ ^]^ In order to effectively conceive novel luminophores, knowledge of the key challenges and the strategies to surpass them is vital.

Generally, an electronically excited molecule rapidly dissipates the energy via vibrational relaxation (VR) and internal conversion (IC) until it reaches the lowest singlet excited state (S_1_).^[^ ^6^ ^]^ *Kasha's* rule states that fluorescence, i.e., radiative deactivation by photon emission, most likely occurs from this state.^[^ ^7^ ^]^ However, fluorescence competes with nonradiative deactivation via IC or collisions with surrounding molecules, as well as intersystem crossing (ISC), which is the transition from singlet to triplet spin multiplicity. In the triplet manifold, relaxation to T_1_ is once again succeeded by a competition between spontaneous emission (phosphorescence), nonradiative deactivation, and reverse intersystem crossing (rISC) back into the singlet state. The latter is an energetically uphill process that opens the path to thermally activated delayed fluorescence (TADF).^[^ ^6^ ^]^

Emission from the triplet state of pure organic compounds remains a challenge today due to the low ISC rates and the rapid nonradiative deactivation of the triplet exciton. As a result, various strategies have been reported to obtain phosphorescent properties in organic compounds.

First observed by *Kasha* and later highlighted by *El‐Sayed*, the radiative transition from T_1_ to S_0_ is enhanced when the participating orbitals have different electronic origins.^[^ ^7^ ^]^ For example, the transition of (n,π*) → (π,π*) is approximately three orders of magnitude faster than for transitions of the same electronic species. This phenomenon is also known as the *El‐Sayed* rule.^[8]^ *Kasha* observed this behavior for *N*‐heterocycles and carbonyl compounds, demonstrating that these moieties can enhance ISC rates.^[^ ^7^ ^]^ As result, these functionalities are often used to receive phosphorescence in organic compounds.

Independent of the participating orbitals, a small energy gap (Δ*E*_ST_) is essential for efficient transition from S_1_ to the triplet manifold, resulting in phosphorescence. This is also crucial for future applications in optoelectronic devices.^[^ ^9^ ^]^

Therefore, not only is the ISC rate important, but the relationship between radiative (*k*_r_) and non‐radiative (*k*_nr_) rates from the populated triplet state is also crucial for achieving phosphorescence. To manipulate the balance between these two deactivation processes, the phosphor can be embedded in a rigidified environment such as a host material or the solid state. This suppresses non‐radiative deactivation, additionally enabling the surrounding molecules to stabilize the triplet exciton, resulting in improved radiative relaxation. For instance, when embedded in a polymer, the phosphor can also be shielded from atmospheric triplet oxygen, thus reducing non‐radiative deactivation.^[^ ^10^ ^]^

Another important strategy for efficient ISC is integrating a heavy atom. The element enhances spin‐orbit coupling (SOC), which increases with atomic number and plays a role in the ISC process. The heavy atom both facilitates the ISC and aids in relaxation from T_1_ to S_0_. This leads to better triplet formation but also shorter lifetimes, as the spin‐forbidden transition resulting in phosphorescence is accelerated as well.^[^ ^7^, ^11^ ^]^

Over the course of the last decades, various structural motifs for efficient organic luminophores have emerged, which can be adapted or combined in countless ways. Amongst these is the popular fused ring system of dibenzofuran (DBF) and dibenzothiophene (DBT), which deserves special attention due to its beneficial properties. These include the extensive delocalized π‐system that offers many possibilities for its synthesis and post‐functionalisation,^[^ ^12^ ^]^ the high‐lying triplet energies^[^ ^13^ ^]^ and excellent charge transport properties.^[^ ^14^ ^]^ Additionally, the moiety is capable of efficient ISC^[^ ^15^ ^]^ and can be utilized either as an electron donor or acceptor, which is why it is frequently combined with other motifs for charge transfer (CT) systems.^[^ ^16^, ^17^ ^]^ Comparing DBF and DBT derivatives, the sulfur congeners typically exhibit better ISC rates based on the heavy atom effect, hence it qualifies as a central motif in phosphorescent luminophores. According to this, incorporating the heavier homologues (Se, Te) into this motif has only been scarcely investigated but may offer the potential to receive improved triplet formation and efficient phosphors.

For this reason, this mini‐review is dedicated to the photoluminescence properties of the whole family of dibenzo[*b,d*]chalcogenophenes (DBC). Our aim is to summarize the recent accomplishments of DBC luminophores comprehensively and to provide insights for future developments. Besides the oxygen and sulfur variants DBF and DBT, of which a large number of luminescent examples are known, we also look into the rarely described homologues dibenzoselenophene (DBSe) and ‐tellurophene (DBTe). To this end, we discuss the challenges when designing luminophores with the heavy congeners.

This work is limited to small molecular emitters, since they offer advantages like simple synthetic routes and facilitated uptake into cells.^[^ ^18^ ^]^ In contrast to polymeric luminophores, problems regarding polydispersity or regioregularity are avoided. For the sake of understanding the complex underlying processes in the aromatic systems in detail, molecules with sizes up to 10 rings and only one chalcogenophene unit are considered. In addition, we set the focus on single‐component systems instead of co‐crystals, host‐guest systems, or composite materials such as OLEDs. Regarding matrices, we consider physical distribution of luminophores in conventional polymers as a medium in which the emission still arises from single molecules, exactly like in solutions or frozen matrices. Hence, polymer matrices are included.

### 1.1 Synthesis

DBF was first discovered in 1866 by *C. Lesimple*, who heated triphenyl phosphate with calcium oxide.^[^ ^19^ ^]^ While he initially assumed to have obtained diphenyl ether, *W. Hoffmeister* elucidated the correct nature of the compound's structure in 1871.^[^ ^20^ ^]^ Around the same time, *J. Stenhouse* described the first synthesis of DBT by oxidizing diphenyl sulfide with hot iron nails.^[^ ^21^ ^]^ Peculiarly, *Stenhouse* also misjudged the product's structure, which was later corrected by *C. Graebe*, the supervisor of *Hoffmeister*.^[^ ^22^ ^]^ The occurrence of both compounds in stone coal tar was discussed as early as 1890,^[^ ^23^ ^]^ whereas experimental proof was delivered in 1901 for DBF^[^ ^24^ ^]^ and in 1920 for DBT.^[^ ^25^ ^]^

DBSe^[^ ^26^ ^]^ and DBTe^[^ ^27^ ^]^ were both synthesized in the 1930s by *C. Courtot*, by dehydration of diphenyl selenoxide with sodium amide and by reaction of biphenyl with tellurium tetrabromide, respectively.

While both DBF and DBT can be obtained from natural sources such as crude oils and coals,^[^ ^28^ ^]^ derivatives of DBF are also found in plants and funghi, some of which are toxicologically active.^[^ ^29^ ^]^ DBSe and DBTe, on the other hand, are exclusively available through synthesis. The synthetic approaches to DBF^[^ ^30^ ^]^ and DBT^[^ ^31^ ^]^ are comprehensively described in recent review articles. For DBSe, *Murphy* compiled known syntheses until 2001,^[^ ^32^ ^]^ while another recent review by *Ma* et al. covers DBSe syntheses using elemental selenium.^[^ ^33^ ^]^ *Sadekov* et al. highlighted synthetic routes to DBTe until 2007.^[^ ^34^ ^]^ In comparison, there is less literature on DBSe and DBTe syntheses than for DBF and DBT.

**Figure 1:**
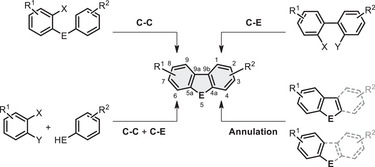
Overview of different synthetic approaches to the DBC motif and numbering convention (E = O, S, Se, Te).

In general, the major approaches to build the tricyclic skeleton can be categorised as follows: C─C coupling from diphenyl chalcogenides, C─E coupling from biaryls, C─C and C─E coupling from two benzenes and annulation from benzochalcogenophenes or phenyl chalcogenols (Figure 1). Naturally, there are also reactions that follow other strategies, like the hexadehydro‐Diels‐Alder reaction with tetraynes to yield fused DBT or DBSe.^[^ ^35^ ^]^ To obtain the heavier DBCs, an element exchange reaction starting from diaryl iodonium salts was reported, especially successful for a series of DBTe compounds.^[^ ^36^, ^37^ ^]^

To obtain functionalized DBCs, the substituents can either be integrated in the precursors or introduced to the complete motif afterwards. Starting from unfunctionalized DBCs, electrophilic aromatic substitution is preferably directed to the 2‐ and 8‐positions.^[^ ^38^, ^39^ ^]^ By manipulation at the chalcogen, for example, oxidation of the sulfur or selenium, the regioselectivity can be diverted to favor positions 3 and 7^[^ ^40^ ^]^ or 4 and 6.^[^ ^41^ ^]^ Additionally, C4 and C6 are accessible via *ortho* lithiation.^[^ ^42^ ^]^ Regioselective substitution of positions 1 and 9 proves to be challenging, hence this substitution pattern is often obtained by separating isomeric mixtures, for example, via Friedel‐Crafts reaction.^[^ ^16^, ^43^ ^]^

Regarding the photophysical properties, it is essential to state whether the DBC moiety was obtained from commercial sources or synthesized and how it was purified, which will be discussed in Section 2.6.

### 1.2 Historical Background of Emission

Phosphorescence properties of DBF and DBT were first reported by *Heckman* in 1958.^[^ ^44^ ^]^ Therefore, the author measured the phosphorescence spectra of different derivatives of fluorene, carbazole (Cz) and other heterocycles at 77 K. The author observed phosphorescence in the blue region at around 410 nm for DBF, whereas the emission seemed to be bathochromically shifted for DBT. Due to the limitation of measurement techniques at that time, the emissions were subjectively described, stating that DBT showed the strongest emission compared with DBF, Cz, and fluorene. As *Kasha* already observed,^[^ ^7^ ^]^ *Heckman* visually verified the trend of shorter afterglow times induced by heavy atoms (∼5 s (DBF), ∼2 s (DBT)).^[^ ^44^ ^]^

Building on this, in 1989, *Zander* and *Kirsch* conducted fluorescence and phosphorescence studies of all benzo[*b*]chalcogenophenes and DBCs (O‐Te) in ethanol at 77 K to investigate the heavy atom effect.^[^ ^11^ ^]^ The emission wavelengths were bathochromically shifted with respect to the increasing size of the incorporated chalcogen (see Table 1). Whereas all homologues showed phosphorescence, for benzo[*b*]tellurophene and DBTe, no fluorescence could be measured. This was attributed to the efficient triplet formation ($\Phi_{T}$) facilitated by improved SOC, also leading to a fast deactivation from T_1_ to S_0_. The oxygen congener DBF exhibited a moderate fluorescence accompanied by a weaker phosphorescence. With the increasing size of the chalcogen (O‐Te), the triplet formation $\Phi_{T}$ seemed to become more efficient, resulting in almost solely phosphorescence for DBSe and DBTe with *Φ*_P_ above 70%. As already mentioned above, despite this, the lifetimes of DBSe and especially DBTe are drastically decreased to 2.5 ms in contrast to the lighter congeners, demonstrating an increased ISC rate for triplet formation as well as for non‐radiative deactivation of the exciton with increasing atomic number.

**Table 1:** Fluorescence and phosphorescence emission maxima *λ* in nm, quantum yields *Φ*, phosphorescence lifetimes *τ*_p_ in s, triplet formation efficiencies *Φ*_T_ and rate constants *k* for radiative and non‐radiative decay for DBCs (O‐Te) measured in ethanol at 77 K by *Zander* and *Kirsch*.^[^ ^11^ ^]^

	DBF	DBT	DBSe	DBTe
*λ*_F_ [nm]	302	329	337	‐
*λ*_P_ [nm]	409	411	417	425
*Φ*_F_	0.4	0.025	0.001	‐
*Φ*_P_	0.29	0.47	0.74	0.79
*τ*_p_ [s]	5.6	1.5	0.04	0.0025
*Φ*_T_	0.6	0.97	≈1	≈1
*k*_r_ [1/s]	0.086	0.32	18.5	316
*k*_nr_ [1/s]	0.092	0.35	6.5	84

Based on the results of *Zander*, *Kirsch*,^[^ ^11^ ^]^ and *Heckman*,^[^ ^44^ ^]^ DBCs present an interesting series of organic compounds exhibiting promising phosphorescent properties at 77 K, leading to their versatile functionalisation and investigation in the following decades in the field of achieving RTP emitters.

## 2 Emission of Dibenzochalcogenophenes

According to the general strategies discussed in the introduction, several functional groups and structural motifs have emerged that efficiently fulfil these requirements. Since the properties and use of the luminophores are strongly dictated by the functionalisation, the main part of this review is structured along the functional groups attached to the DBCs. After a brief summary of unfunctionalized DBCs, we will discuss DBCs with carbonyl and sulfonyl groups, nitrogen heterocycles and amines, combinations thereof and other functional groups.

*Fang* and *Yan* reported RTP properties of unsubstituted commercial DBT in single crystals.^[^ ^45^ ^]^ They obtained two polymorphs, a block‐shaped cyan‐emitting (α) and a rod‐shaped crystal (β). The latter displayed white light emission with *Commission Internationale de l'Eclairage* (CIE) coordinates of (0.329, 0.336) under UV light and significant visible yellow afterglow. The phosphorescence emission was composed of two bands at 460 and 570 nm with lifetimes of 590 µs and 124 ms, respectively. The authors explained the RTP with favorable face‐to‐face packing of DBT units, enabling π‐π‐stacking.

Similarly, *van Hecke* and coworkers reported RTP for single crystals of commercial DBT (named **DBS** in the publication) and 2‐bromo‐DBT (**DBS‐Br**) with lifetimes in the microsecond regime.^[^ ^46^ ^]^ While dual emission of fluorescence (356 nm) and phosphorescence (563 nm) was observed for **DBS**, **DBS‐Br** presented solely blue phosphorescence (441 nm). Moreover, the single crystals were elastically flexible in one or two dimensions. Both phenomena were accredited to chalcogen‐centred weak interactions such as σ/π‐hole type S···π and C‐H···S.

*Zhang* et al. optimized the RTP of commercial DBF and DBT by incorporating them in a polyvinyl alcohol (PVA) matrix.^[^ ^47^ ^]^ At an optimum concentration of 1 mg/mL, they achieved astounding phosphorescence lifetimes of 1568 and 700 ms under ambient conditions for DBF and DBT, respectively. The RTP was well enhanced in comparison to the crystalline compounds, which was attributed to hydrogen bonds in the matrix providing a rigid environment and shielding from oxygen and moisture. Additionally, the doped films were largely flexible, which enabled the fabrication of 3D folded or bent objects.

### 2.1 Carbonyl and Sulfonyl Groups

The combination of the DBC motif with carbonyl groups is favorable due to the availability of lone pairs and the donor/acceptor character of the two building blocks. Most reports feature an aldehyde or ketone in 2‐position, which is synthetically easily accessible.

**Figure 2:**
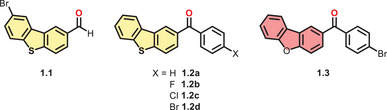
Structural formulas of presented carbonyl substituted DBCs.

*Li* et al. replaced the nitrogen in an aldehyde‐substituted carbazole (Cz) with sulfur to obtain the DBT **1.1** (Figure 2).^[^ ^48^ ^]^ Although both compounds displayed RTP in the solid state, the relative emission intensities revealed that the overall emission of the Cz was mainly caused by fluorescence. Conversely, phosphorescence prevailed in the case of **1.1**, which was attributed to dense H‐aggregates in the crystal structure. Additionally, two polymorphs, **1.1α**, and **1.1β**, were shown to have excitation‐dependent emission, which influences the relative intensities of fluorescence and phosphorescence. The authors explained this with the existence of two kinds of emitting species, which results in multiple competing ISC channels.

Earlier, *Tang* and coworkers had reported DBTs that were inherently fused with the benzophenone motif (**1.2a‐d**, Figure 2).^[^ ^49^ ^]^ Due to the contribution from the oxygen lone pairs and the shared conjugated system of the DBT and phenyl groups, dual RTP in crystals originating from the first and second excited triplet state was achieved. In this case, phosphorescence from T_2_ can compete with T_1_ because of its (n,π*) transition character, which grants larger SOC according to the *El‐Sayed* rule. For compound **1.2c**, the combination of short‐lived (0.41 ms) blue phosphorescence and long‐lived (123.4 ms) yellow phosphorescence (Table 2) produced white light emission (CIE 0.33, 0.35).

Later, *Chen* et al. discovered that the DBF derivative of **1.2d** (**1.3**, Figure 2) also exhibits RTP with lifetimes up to 104 ms in single crystals (Table 2).^[^ ^38^ ^]^ They reported two polymorphs showing temperature‐sensitive phosphorescence. Polymorph **1.3α** revealed weak RTP, but a 40‐fold luminance increase upon lowering the temperature to 77 K, caused by reduced thermal motion and vibrations. On the other hand, **1.3β** featured bright yellow RTP that turned bluish green at 77 K, which was assigned to an enhanced emission from the triplet locally excited (^3^LE) state. Due to the versatile temperature responses, the authors proposed possible applications as temperature sensors.

**Table 2:** Phosphorescence emission maxima *λ*_P_, lifetimes *τ*_P_ and photoluminescence quantum yields *Φ*_PL_ of carbonyl‐substituted DBCs in crystalline solid‐state at RT.

Compound	*λ*_P_ [nm]^[a]^	*τ*_P_ [ms]^[a]^	*λ*_P_ [nm]^[b]^	*τ*_P_ [ms]^[b]^	*Φ*_PL_ [%]	Refs.
**1.1α** ^[c]^	‐	‐	535	0.68	<0.1^[e]^	[48]
**1.1β** ^[d]^	‐	‐	555	0.72	0.7^[e]^	[48]
**1.2a**	470	0.71	567	103.7	2.3	[49]
**1.2b**	465	0.062	565	106.7	6.5	[49]
**1.2c**	470	0.41	590	123.4	7.2	[49]
**1.2d**	467	0.14	570	103.8	6.8	[49]
**1.3α**	‐	‐	567	67.22	4	[38]
**1.3β**	‐	‐	560	91.90	25	[38]

^[a]^ Emission from T_2_.

^[b]^ Emission from T_1_.

^[c]^ Properties upon excitation at 380 nm.

^[d]^ Properties upon excitation at 365 nm.

^[e]^ Phosphorescence quantum yield.

**Figure 3:**
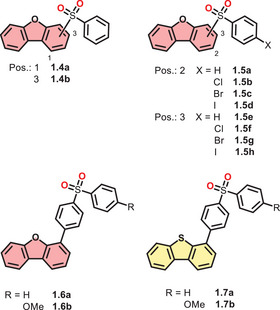
Structures of DBCs functionalized with sulfonyl groups and discussed with respect to their photophysics.

In addition to carbonyl‐substituted DBCs, sulfones represent another main motif, which is reported frequently in recent literature (see Figure 3). Based on the available lone pairs and the incorporation of another heavy atom (sulfur), phosphorescence properties should be obtained and possibly even enhanced for longer lifetimes (following *El‐Sayed*’s rule).^[^ ^50^ ^]^

*Chen* et al. introduced a sulfonyl group to DBF in 1‐ and 3‐positions (**1.4a,b**, Figure 3), achieving delayed emission and RTP properties. The 1‐regioisomer (**1.4a**) exhibited a bathochromic shift in UV/vis and PL spectra due to higher steric hindrance. Both compounds showed significant multi‐emission in the crystalline state (**1.4a**: two delayed bands; **1.4b**: three delayed bands, Table 3). The hypsochromic emission band (*λ*_em,**1.4a** _ =  423 nm; *λ*_em,**1.4b** _ =  403 nm) stems from delayed fluorescence based on intermolecular interactions in the crystal lattice. The delay of the fluorescence can be observed at room temperature, but the lifetime decreases with lower temperature. Based on theoretical calculations, the authors explain this behavior according to a large energy gap between the S_1_ and T_1_ state, ruling out TADF, leading to the assumption, that triplet‐triplet‐annihilation (TTA) is responsible for the delay. The TTA process is accompanied with the dimer formation of the molecules via π−π stacking in dense packing, facilitating energy transfer.

The second delayed band, around 500 nm, was attributed to monomeric phosphorescence induced through the DBF core only, verified by measurements in 2‐methyltetrahydrofuran as glassy matrix at 77 K, which showed the same emission for both regioisomers **1.4a** and **b**.

For **1.4b**, the TTA process is accompanied by the third emission band at 586 nm with a lifetime of 230 ms, which is also increasingly observed in concentrated solution. This can be explained by facilitated ISC, leading to a decrease of fluorescence and the occurrence of the new phosphorescence band (Table 3).^[^ ^43^ ^]^

**Table 3:** Fluorescence and phosphorescence emission maxima *λ* in nm, phosphorescence lifetimes *τ*_p_ and photoluminescence quantum yields *Φ*_PL_ for the presented sulfonyl‐substituted DBCs for crystalline samples at RT.

Compound	*λ*_F_ [nm]	*λ*_P_ [nm]	*τ*_p_ [s]	*Φ*_PL_	Refs.
**1.4a**	372 403^[a]^	563	0.242	0.46 0.15^[b]^	[43]
**1.4b**	352 423^[c]^	510 586	0.498 0.230	0.55 0.26^[b]^	[43]
**1.5a**	425	550	0.027	0.22	[16]
**1.5b**	430	550	0.150	0.19	[16]
**1.5c**	425	540	0.164	0.20	[16]
**1.5d**	435	550	0.014	0.02	[16]
**1.5e**	430	560	0.075	0.12	[16]
**1.5f**	480	580	0.072	0.18	[16]
**1.5g**	425	560	0.084	0.16	[16]
**1.5h**	425	550	0.031	0.04	[16]
**1.6b**	372	476 580	0.204^[d]^ 0.627^[d]^	0.62^[d]^	[51]
**1.7a** ^[e]^	386	519	0.036	0.07	[52]
**1.7b**	377	563	0.171	0.05	[53]

^[a]^ Delayed fluorescence (*τ*_DF_ **= 78.2 µs).

^[b]^ Quantum yields of delayed fluorescence and phosphorescence.

^[c]^ Delayed fluorescence (*τ*_DF_ **= 282.5 µs).

^[d]^ Measured in helium atmosphere.

^[e]^ Ground sample.

Introducing also the 2‐sulfonylphenyl‐substituted DBFs with halogens on the benzenesulfonyl residues, *Voskuhl* and coworkers investigated a series of DBF derivatives (**1.5** **a‐h**, Figure 3), observing similar emission behavior. Emission occurred via intramolecular CT from the DBF cycle to the sulfonyl group, showing better emission when the latter is in 2‐ or 3‐position, which could be correlated with the stacking motif inside the crystal lattice. Comparing different halogens in the *para‐*position on the benzenesulfonyl moiety, chlorine, and bromine (**1.5b**, **1.5f**, **1.5c**, **1.5g**) derivatives exhibited *Φ*_PL_ up to 22% in the crystalline state. Besides efficiency, compounds **1.5b** and **1.5c** show the longest lifetimes, about 150 ms, among the studied library (Table 3). This regiospecific luminescence correlates with strong π−π stacking according to scXRD (single crystal X‐ray diffraction) analyses. In contrast to the chlorine derivative, the bromine congeners showed slightly worse emission properties. This trend continued for the iodine compounds (**1.5d**, **1.5h**), explained by stronger halogen‐driven interactions in the crystal packing and SOC favoring non‐radiative pathways. Due to combination of the emission bands in the blue and yellow region for the bromine congeners (**1.5c**, **1.5g**), the authors were able to achieve white light emission (CIE: 0.29, 0.35, and 0.31, 0.34).^[^ ^16^ ^]^

Arguing with intermolecular interactions, *Chen* et al. introduced sulfonyldiphenyl‐substituted DBF in 4‐position, alternating between no residue and a methoxy group (**1.6a**/**b**, Figure 3).^[^ ^51^ ^]^ Therefore, the latter showed additional emission behavior through TADF via intermolecular CT by inducing intermolecular hydrogen bonding. The authors also reported green persistent RTP (*τ*_pRTP_  =  627 ms) in crystalline sample for **1.6b** from the DBF core, whose emission is enhanced by stronger π‐π stacking and hydrogen bonding in the crystal lattice compared to **1.6a**, which yields a total *Φ*_PL_ of 62% for the crystalline sample. The authors explain this by denser packing and therefore suppression of nonradiative decay pathways inside the crystalline state.^[^ ^51^ ^]^

In 2017, *Xu* et al. substituted the oxygen with sulfur in the DBC core of the above‐mentioned motif of *Chen* et al. (**1.6a**) to obtain **1.7a**.^[^ ^52^ ^]^ In the crystalline solid at room temperature, they observed dual emission comprising blue fluorescence and yellow phosphorescence (*τ*_P_ = 35.6 ms), achieving white light emission with CIE coordinates of (0.24, 0.26). The authors attributed the phosphorescence to efficient SOC at the sulfonyl oxygens, as well as a moderate Δ*E*_ST_ and strong hydrogen bonds between the DBT core and sulfonyl oxygen. Thus, the phosphorescence could be diminished by grinding, due to a planarization of the system, thereby only the fluorescence emission remains at 386 nm in the deep blue region. Upon fuming with DCM vapor, the crystalline structure and phosphorescence were restored, showing the mechanochromism of compound **1.7a**.

The group of *Chen* et al. published the same molecule with a DBT core, but methoxy‐functionalized (**1.7b**). The sulfur congener exhibited two emission bands in crystalline solid, with one attributed to fluorescence (*λ*_em _ =  389 nm; *τ*_F_  =  3.8 ns) and the other to monomeric phosphorescence (*λ*_em _ =  563 nm; *τ*_P_  =  171 ms). By theoretical calculations, the authors were able to resolve the ISC channels. Thereby, the monomeric compound showed only one channel, although the dimer, formed by hydrogen bonding induced by the methoxy group, exhibited four ISC channels. Additionally, the triplet energy was lowered, leading to long‐lived phosphorescence through this interaction, which restricts molecular motion. Therefore, the authors were able to demonstrate the high influence of the packing inside the crystal structure on the RTP properties in these molecules.^[^ ^53^ ^]^

### 2.2 Nitrogen‐Containing Heterocycles and Amines

Unlike in carbonyl‐ or sulfonyl‐substituted DBCs, the DBC rather acts as an electron acceptor when connected to nitrogen‐containing heterocycles.

**Figure 4:**
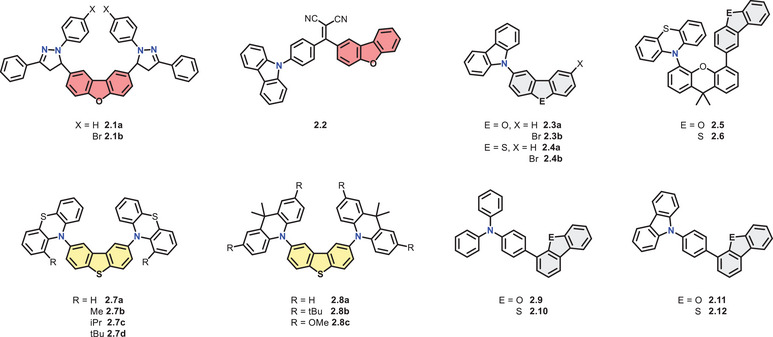
Structural formulas of presented DBCs which are substituted with nitrogen heterocycles and amines.

In 2011, the group of *Fang* presented two pyrazoline derivatives based on DBF, one of which was brominated at the side chain (**2.1a‐b**, Figure 4).^[^ ^17^ ^]^ The authors examined the emission solely in solution, which was determined to be pure fluorescence with good quantum yields of up to 30%. Here, the DBF served as an electron acceptor and the pyrazoline as a donor, albeit only a small solvatochromic shift was observed. The overall small Stokes shift of the compounds indicates the rigid molecular geometry. By theoretical analysis, the authors showed that the compounds could qualify as hole transport materials in OLEDs due to low hole reorganisation energies.

*Zhan* and coworkers described a D‐A‐D' type compound (**2.2**, Figure 4) consisting of Cz and DBF bridged by a dicyanovinyl unit, which led to more pronounced solvatochromism.^[^ ^54^ ^]^ Due to the ICT character between the Cz and the dicyanovinyl moiety, the fluorescence emission wavelengths spanned from 513 to 615 nm between toluene and acetone. Moreover, the twisted conformation of the molecule resulted in relatively loose packing in the solid state, which facilitated the mechanochromic properties of **2.2**. In reversible grinding‐fuming cycles, the fluorescence was tuned from bright yellow (*λ*_em_ = 525 nm, *Φ*_PL _= 39%) to orange (*λ*_em _ =  558 nm, *Φ*_PL _ =  15%).

In 2019, the groups of *He* and *Tang* used a simpler system to exemplify an important design strategy that resolves the conflict between long lifetimes and high phosphorescence quantum yields.^[^ ^15^ ^]^

Achieving persistent phosphorescence requires a slow radiative decay (*k*_r_). However, the longevity of the triplet state renders it prone to nonradiative deactivation (*k*_nr_) and quenching (*k*_q_), reducing the quantum yield. At the same time, efficient ISC correlates with shorter lifetimes due to high *k*_r_, *k*_nr_, and possible rISC. Thus, long lifetimes and high phosphorescence quantum yields generally conflict with each other.

In their work, *He* and *Tang* described that DBF and DBT enable efficient ISC but suffer from short lifetimes, while Cz provides long‐lived triplet states and poor ISC.^[^ ^15^ ^]^ Thus, they integrated both moieties in close proximity (**2.3a‐b**, **2.4a‐b**, Figure 4) to enable a Dexter‐type triplet‐triplet energy transfer (TTET). Promoted by a small energy gap and a heavy bromine atom, efficient ISC at the DBC site was followed by nearly quantitative intramolecular TTET to the Cz unit. Consequently, high *Φ*_PL_ values of 10–41% combined with impressive lifetimes in the range of 420–652 ms were achieved (Table 4).

**Table 4:** Fluorescence (F) and phosphorescence (P) emission maxima *λ*, lifetimes *τ* and quantum yields *Φ* of DBCs with nitrogen heterocycles and amines in crystalline solid‐state at RT.

Compound	*λ*_F_ [nm]	*τ*_F_ [ns]	*Φ*_F_ [%]	*λ*_P_ [nm]	*τ*_P_ [ms]	*Φ*_P_ [%]	Refs.
**2.2**	525	5.96	‐	‐	‐	‐	[54]
**2.3a**	411	12.2	43	549	652	14	[15]
**2.3b**	411	8.0	32	550	540	41	[15]
**2.4a**	413	12.3	28	551	450	10	[15]
**2.4b**	413	8.1	22	551	420	12	[15]
**2.5**	444	1.03	2.02^[a]^	510	1.12	‐	[55]
**2.6**	433	1.41	4.25^[a]^	514	5.00	‐	[55]
**2.9**	429	‐	13.1	550	114	1.3	[57]
**2.11**	414	‐	35.2	550	395^[b]^	0.5	[57]
**2.12**	414	‐	9.2	550	229^[b]^	0.6	[57]

^[a]^ Combined *Φ*_L_ of fluorescence and phosphorescence

^[b]^ Enhanced by impurity–see chapter 2.6.

Similarly, the groups of *Li* and *He* presented a strategy to enable through‐space SOC, whereby the D and A segments were connected in spatial vicinity, but with weak electronic coupling.^[^ ^55^ ^]^ In contrast to CT systems that grant spatial HOMO‐LUMO separation (and thus small Δ*E*_ST_) by strong conjugated donors and acceptors, here DBF/DBT and phenothiazine (PTZ) were bridged by an unconjugated 9,9‐dimethylxanthene (**2.5**–**2.6**, Figure 4). The crystal structures revealed a face‐to‐face alignment of the DBC and PTZ rings with distances of 3.34–3.60 Å. The resultant emission of both compounds in the crystalline solid was dominated by phosphorescence, which was attributed solely to the PTZ unit. Although the *Φ*_PL_ of 2–4% and lifetimes of 1–5 ms were not large in relation to known phosphors, the authors proposed high versatility of this approach. Since there is no need for significant conjugation and strong donor or acceptor capabilities, it was suggested that the three building blocks could be tuned quite freely.

Moreover, phenothiazine can adopt an H‐intra (quasi‐equatorial) or H‐extra (quasi‐axial) conformation, which can influence the photophysical properties. *Huang* et al. investigated this with PTZ substituted DBTs (**2.7a‐d**, Figure 4), which show RTP in zeonex films under vacuum.^[^ ^56^ ^]^ To this end, they attached substituents with increasing steric demand (H, Me, *i*Pr, *t*Bu) to the PTZ skeleton. Expectedly, the bulky substituents raised the energy barrier between the H‐intra and H‐extra conformers. First, the unsubstituted **2.7a** exhibited efficient green RTP (*λ*_em_ = 540 nm, *Φ*_L_  =  60%) originating from the low‐energy (equatorial) conformation. In the methyl derivative **2.7b**, relaxation to the low‐energy form is slowed down, which enables dual RTP (*λ*_em _= 450; 540 nm, Table 5). Finally, the larger alkyl substituents lock the molecule in the high‐energy (axial) conformer, resulting only in blue phosphorescence (*λ*_em _= 450 nm).

**Table 5:** Emission maxima *λ*, lifetimes *τ*, and quantum yields *Φ* for fluorescence (F) and delayed luminescence (DL) of DBCs with nitrogen heterocycles and amines in polymer matrices at RT.

Compound	*λ*_F_ [nm]	*τ*_F_ [ns]	*Φ*_F_ [%]	*λ*_DL_ [nm]	*τ*_DL_ [ms]	*Φ*_DL_ [%]	Refs.
**2.7a** ^[a]^	450	12.6	6	540^[b]^	47	60	[56]
**2.7b** ^[a]^	390	0.60	3	450, 540^[b]^	24	1	[56]
**2.7c** ^[a]^	390	0.65	4	450^[b]^	28	2	[56]
**2.7d** ^[a]^	390	0.67	3	450^[b]^	78	2	[56]
**2.8a**	398^[c]^	‐	1	398^[^ ^[d]^, ^[e]^ ^]^	‐	6^[f]^	[58]
**2.8b**	414^[c]^	5.9	20	414^[^ ^[d]^, ^[e]^ ^]^	0.136	54^[f]^	[58]
**2.8c**	460^[c]^	9.6	35	460^[^ ^[d]^, ^[e]^ ^]^	0.011	48^[f]^	[58]
**2.9**	425	2.83	33.3	516^[b]^	770	3.8	[57]
**2.10**	419	2.56	18.1	530^[b]^	260	2.9	[57]
**2.11**	393	6.17	42.1	510^[b]^	1510	1.1	[57]
**2.12**	403	1.98	10.0	514^[b]^	250	0.4	[57]

^[a]^ Measurement under vacuum.

^[b]^ Phosphorescence.

^[c]^ Measurement under air.

^[d]^ Measurement under nitrogen atmosphere.

^[e]^ TADF.

^[f]^ Total *Φ*_PL_ determined under nitrogen atmosphere, consisting of prompt fluorescence and TADF.

Expanding on this, *Huang* et al. replaced the PTZ unit with an acridine moiety, which possesses higher triplet energies akin to DBT and should lower the Δ*E*_ST_ for efficient rISC and TADF.^[^ ^58^ ^]^ For increased stability and donor strength, *t*Bu or OMe groups were added to the acridine core (**2.8a‐c**, Figure 4). In zeonex matrices at RT, the compounds displayed prompt fluorescence under air and a combination of prompt and TADF under nitrogen atmosphere. The *t*Bu derivative **2.8b** featured a narrow Δ*E*_ST_ of 0.09 eV due to a lowered ^1^CT state. Conversely, the OMe groups lowered both the ^1^CT and the ^3^CT state, which resulted in a bigger energy gap of 0.24 eV. As a result, **2.8b** exhibited a higher TADF contribution (*Φ*_PL_ increase of 34% under nitrogen) than **2.8c** (13% increase under nitrogen, Table 5). However, the lifetime of the OMe derivative (10.5 µs) was shorter than for *t*Bu (136.4 µs), indicating fast rISC, which is not expected for a bigger energy gap. The authors proposed that the rISC in **2.8c** occured from the higher ^3^LE instead of the lowest ^3^CT, which explains the lower TADF efficiency and the faster decay. Hence, it was highlighted that the fine‐tuning of triplet states is crucial for designing TADF emitters.

In contrast to the distribution of organic luminophores in preexisting polymers, *Chen* et al. demonstrated efficient stabilisation of triplet states at RT by in situ polymerisation.^[^ ^57^ ^]^ Twisted D‐A compounds composed of triphenylamine (TPA) or Cz and DBF/DBT (**2.9**–**2.12**, Figure 4) were dissolved in methyl methacrylate (MMA) and polymerized to obtain single‐molecular green RTP with astonishing lifetimes of up to 1.51 s (Table 5). This approach yielded longer lifetimes than post‐doping of PMMA with luminophore, indicating better distribution and immobilisation. Moreover, an optimum concentration of 1 wt% was identified. According to their analysis, multiple T_n_ close to S_1_ and the CT character contributed to the efficient emission of the polymer **2.11**.

Interestingly, the single crystal solid‐state phosphorescence of **2.11** was indicated to be enhanced due to small amounts of a Cz isomer. However, the phosphorescence of polymer **2.11** was unaffected when highly pure lab‐synthesized Cz was utilised. This behavior gives a first hint for the role of regioisomer doping, which will be discussed in chapter 2.6.

### 2.3 Combinations

From the scope we presented earlier, some small luminophores described in the literature show combinations of the before‐classified main motifs. Therefore, in the following section, we present some merged structures, which combine, for example, amines with carbonyl functionalities and more. Most of the investigated compounds are based on donor‐acceptor combinations to induce CT emission states.

**Figure 5:**
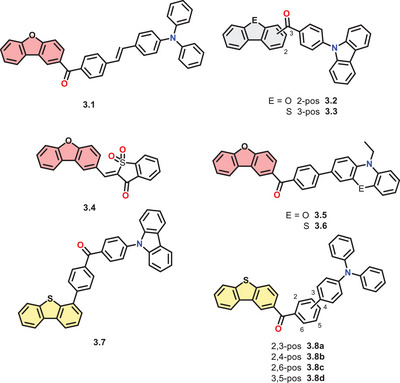
Presented combinations of the functionalities on DBC cores studied for their photophysical properties.

In 2022, *Liu* and coworkers merged a 2‐benzoyl‐substituted DBF core with triphenylamine connected via a styrene (**3.1**, see Figure 5).^[^ ^59^ ^]^ Thereby, the authors yielded a system with a nitrogen‐containing donor, where the HOMO is mostly located, and the carbonyl group with an electron‐accepting LUMO. Based on this structure, intramolecular CT properties can be observed, such as a bathochromic shift of the emission maximum and an increase in the Stokes shift correlating with increasing solvent polarity. In the solid state, three different emission behaviors can be observed. First, the crystalline state shows light blue emission at 465 nm. By grinding, a yellow emission maximum with slightly lower *Φ*_PL_ is obtained (Table 6), probably induced by planarization of the molecule resulting in a larger overlap of the conjugated systems and therefore a red‐shift in emission. Fuming the sample in DCM vapor or annealing leads to a yellow‐greenish emission, showing a different packing pattern according to pXRD (powder X‐ray diffraction). With this, the authors showed stimuli‐responsive solid‐state emission by embedding donor and acceptor units in a large π‐conjugated system.^[^ ^59^ ^]^

**Table 6:** Fluorescence and phosphorescence emission maxima *λ* in nm, lifetimes *τ* and photoluminescence quantum yields *Φ*_PL_ in crystalline state at RT.

Compound	*λ*_F_ [nm]	*τ*_F_ [ns]	*λ*_P_ [nm]	*τ*_p_ [s]	*Φ*_PL_	Refs.
**3.1**	465 (535)	0.43 (3.39)	‐	‐	0.28 (0.23)	[59]
**3.2α**	430	‐	504	0.76	‐	[60]
**3.2β**	512	‐	560	0.44	‐	[60]
**3.2γ**	447	‐	560	0.32	‐	[60]
**3.3** ^[a]^	485	7600	‐	‐	0.25	[61]
**3.3** ^[b]^	440	1.11	465 496 570	0.038 0.113	‐	[61]
**3.5**	471 (575)	2.48 (10.23)	‐	‐	0.34 (0.27)	[63]
**3.6**	482 (564)	3.86 (8.32)	‐	‐	0.39 (0.33)	[63]
**3.7**	422	1.6 11800	569 615	0.415 0.412	‐	[64]

Values in parentheses belong to ground samples.

^[a]^ Emission in dichloromethane solution (20 µM).

^[b]^ Emission at 77 K in dichloromethane solution (20 µM).

Adding a benzoyl moiety to the DBF core in 2‐position and a *para*‐Cz moiety (**3.2**), *Mane* et al. were able to achieve pRTP properties.^[^ ^60^ ^]^ The authors focused on the emission behavior in the aggregated state to hinder non‐radiative decay via rotational or vibrational pathways. From the compound **3.2**, three polymorphs were obtained and investigated separately (**3.2α**, **3.2β,** and **3.2γ,** Table 6). Polymorphs **3.2α**, and **3.2β** exhibited green fluorescence with multiple emission bands followed by a yellow to orange afterglow. The white crystal of **3.2γ** showed a single emission maximum at 447 nm with a short orange afterglow. Quantum chemical calculations were examined to achieve a better insight. It was revealed that the exciton formation in **3.2α**, and **3.2β** is enhanced due to a smaller energy gap based on the formation of dimer‐like structures in the crystal, in contrast to **3.2γ**. This also explains the longer lifetimes of **3.2α** and **3.2β** of around 700 and 450 ms. Additionally, the authors correlate the free volume inside the crystal lattice with the longer lifetimes because dense crystal packing can impede non‐radiative decay by vibrations.

By changing the chalcogen to sulfur and substituting the 3‐position (**3.3**), Fu and coworkers received an organic RTP emitter in solution. Three different emission maxima could be observed, one of those attributed to a short fluorescence band at 440 nm (1.11 ns). A cyan (465 nm, 38 ms) and yellow emission (570 nm, 113 ms) were characterized as phosphorescence. Therefore, the bathochromic band belongs to the transition of T_1_ to S_0_. The authors suggest the other emission originates from a T_2_ to S_0_ transition, supported by quantum chemical calculations. This phenomenon contradicts the *Kasha* rule, which can be explained by a rapid ISC process from S_1_ to T_2_, followed by low IC toward T_1_ but fast radiative decay from T_2_ to S_0_.^[^ ^61^ ^]^

The group of *Sekar* from Mumbai combined a benzo[*b*]thiophen‐3(2H)‐one‐1,1‐dioxide (BTD) with DBF in 2‐position over a C─C double bond, yielding a D‐π‐A‐system (**3.4**). The intramolecular CT character was investigated by its solvatochromism, showing a weaker emission at 430 nm in toluene and a stronger band at 449 nm in DMSO. Using a polar protic solvent, for example, methanol, leads to fluorescence quenching. Introducing Cz or triphenylamine as a donor shifts both absorption and emission toward the red region, whereas the DBF emitter shows the highest Stokes‐shift in all solvents (3554–4829 cm^−1^). In the solid‐state, an emission at 529 nm could be observed. Interestingly, the DBF compound showed an increased quantum yield in polar media (31% in 95/5 H_2_O/ACN), concluding that compound **3.4** is an AIEgen, whereas the Cz compound showed ACQ properties.^[^ ^62^ ^]^

Combining 2‐benzoyl DBF with a phenoxazine/‐thiazine yielded a D‐A‐luminophore (**3.5**, **3.6**), which was investigated by *Zhou* et al.^[^ ^63^ ^]^ With the carbonyl group as the acceptor and the phenoxazine/‐thiazine (POZ/PTZ) as the donor moiety, the luminophore exhibits classical intramolecular CT behavior. By increasing the solvent polarity from hexane (**3.5**: 445 nm; **3.6**: 472 nm) over ethyl acetate (**3.5**: 550 nm; **3.6**: 562 nm) toward DMF (**3.5**: 621 nm; **3.6**: 633 nm), a strong bathochromic shift in emission from blue to dark orange can be observed. This increase in Stokes shift is accompanied by a decrease in *Φ*_PL_, revealing high values in hexane (65–68%) to almost no detectable *Φ*_PL_ in DMF. The authors explain this behavior by the concurrence of simultaneous emission from the LE (locally excited) and CT state in apolar media, whereas in polar media, only the lower‐lying CT state is present, benefiting a nonradiative decay path.

Due to their twisted arrangement, the compounds **3.5** and **3.6** and exhibit strong mechanofluorochromic (MFC) behavior. Microcrystalline powders show bright blue to greenish emission (Table 6). Grinding leads to a shift toward yellow, slightly decreasing the *Φ*_PL_. This is explained by planarization of the structure, leading to a planarized intramolecular CT (PLICT)^[^ ^65^ ^]^ system with an extended conjugational system, exhibiting bathochromic shifted emission. The first state is recovered by exposing the ground sample to DCM vapor, demonstrating a highly reversible MFC, suitable for information storage. The lifetimes of the fluorescence of both solid samples were measured, revealing a slightly longer lifetime for the powders in contrast to the microcrystalline solids. The authors attribute this to different molecular conformations and stacking modes inside the solid affected by the grinding.^[^ ^63^ ^]^

By substituting a benzophenone with either DBT or Cz on each side, *Huang* et al. obtained a D‐A‐D’‐luminophore (**3.7**).^[^ ^64^ ^]^ The solvatochromism showing the CT character of the compound could be observed in solvents of different polarities. In apolar hexane, toluene, and THF, two emission maxima were observed, corresponding to LE states from the DBT and Cz moieties (∼410, ∼430 nm). In polar solvents, both LE and intramolecular CT emission were observed, hinting toward the existence of hybridized local and charge transfer (HLCT) excited states.

The solid‐state samples showed multiple emissions. Under UV light, purple emission could be obtained, followed by an orange afterglow lasting more than 1 s. Three main emissions could be measured in delayed PL spectra. The prompt emission band at 422 nm is ascribed to fluorescence, whereas the same emission is shown in delayed emission spectra with a lifetime of 11.8 µs, revealing its DF character. TADF was ruled out due to constancy in temperature‐dependent measurements (77–300 K), leading to the assumption that this emission is generated by triplet‐triplet‐annihilation (TTA). Additionally, the authors report two phosphorescence bands at 569 and 615 nm with a lifetime above 400 ms, yielding pRTP. The intensity of these triplet emission bands could be increased by submitting the solid to vacuum, impeding quenching via oxygen. By examining in‐depth crystal packing analysis and TD‐DFT calculations, the authors suggest that the RTP originated from the CT from the Cz toward the carbonyl group enabled by intermolecular interactions in the crystal. The large transition dipole moment of the dimer, which was formed by these interactions, plays a crucial role in stabilizing the triplet exciton. This theory could be verified by dual‐mode mechanochromism. The violet emission of the original solid with orange afterglow turned into a dark blue emission after grinding, completely erasing the phosphorescence. The phosphorescence in the solid‐state mainly originates from multiple interacting molecules, stabilizing triplet emission.^[^ ^64^ ^]^

As presented by the above, the combination of donor and acceptor moieties enables delayed emission behavior such as TADF and phosphorescence. By this, these compounds qualify for electronic applications, which was demonstrated by *Ghasemi* et al.^[^ ^66^ ^]^ The authors modified 2‐benzoyl DBT with triphenylamine residues on the benzoyl ring (**3.8**) and obtained four different regioisomers, alternating the substitution pattern. In dependence on this, the compounds were suitable for application in OLEDs, where the 2–4‐regioisomer (**3.8b**) showed a green emission with an external quantum efficiency of 14%. Even in a host material (Zeonex), the compound **3.8d** could be used for oxygen sensing with good sensitivity. This demonstrates the outstanding potential of these motifs, especially for optoelectronic applications.

### 2.4 Other Functional Groups

Besides the frequently recurring motifs found in the literature above, there are also occasional reports with less common structural motifs.

**Figure 6:**
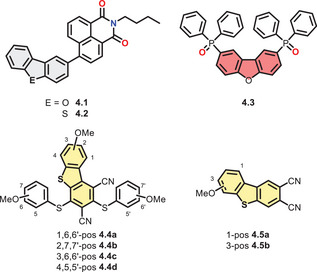
Structural formulas of presented DBCs with other functional groups and motifs.

*Liang* et al. investigated twisted dyads consisting of DBF/DBT as donor and naphthalimide as acceptor connected by a single bond (**4.1**/**4.2**, Figure 6).^[^ ^67^ ^]^ In solvents of low polarity, ISC occurred via spin‐orbit induced (n,π*) → (π,π*) transition. Higher solvent polarities shifted the ISC pathway to spin‐orbit charge transfer (SOCT‐ISC), whereby charge separation (CS) and subsequent charge recombination (CR) enabled triplet state population. The CS state helps to cross the energy gap to the triplet state. In direct comparison, 4.2 achieved higher singlet oxygen quantum yields (75% versus 58%) due to faster CS.

*Vecchi* et al. modified DBF with diphenylphosphine oxide in 2‐ and 8‐position in order to enhance its CT properties while retaining the high triplet energy (**4.3**, Figure 6).^[^ ^68^ ^]^ In CH_2_Cl_2_ solution at room temperature, an impressive fluorescence quantum yield of 44% versus quinine sulfate was determined. Upon freezing the solution at 77 K, phosphorescence with a lifetime of 3.05 s emerged, which was facilitated by SOC with the phosphorus atoms. Due to the ‐I effect of the P = O moieties, the LUMO was lowered, raising electron injection and transport capabilities in OLEDs.

*Haberhauer*, *Strassert*, and *Voskuhl* described substituted DBTs that were synthesized by photochemical ring closure and hence easily gave access to high degrees of substitution.^[^ ^69^ ^]^ The products featured push‐pull systems due to nitrile, arylthiol‐ and methoxy‐groups (**4.4**–**4.5**, Figure 6). While high *Φ*_PL_ of 95% and long lifetimes of up to 890 ms were achieved at 77 K, the unique feature lies in their in situ synthesis in materials. By incorporating the precursor in PMMA films or 3D‐printed objects, the generation of the DBT was induced by photoirradiation, which activates phosphorescence. This way, the materials were inscribed with phosphorescent motifs.

### 2.5 Selenium and Tellurium

**Figure 7:**
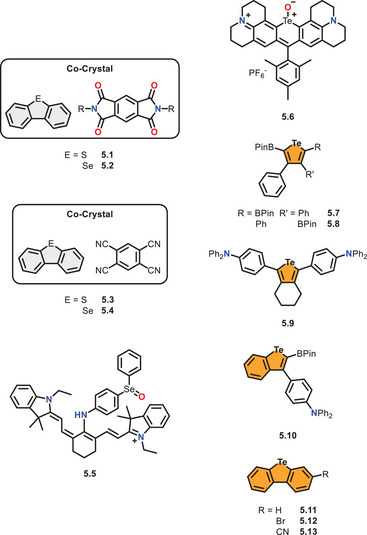
Reported selenium‐ and tellurium‐containing emitters or systems discussed in this section.

In contrast to their lighter homologues, DBSe and ‐Te have scarcely been reported in recent literature, especially for luminescent applications. Most publications present new synthetic procedures or applications in terms of supramolecular assemblies. This lack of information may be due to impeded synthetic accessibility or toxicity. Despite this, the section aims to present a few works that focused on DBSe and ‐Te compounds in terms of photophysics. Additionally, some studies outside the scope presented before will be highlighted to give insight into the recent state of the art (Figure 7).

Besides the reported luminescent properties by *Zander* et al. in 1989,^[^ ^11^ ^]^ DBSe has only been used for cocrystallization experiments with electron‐deficient benzenes like tetracyanobenzene or pyromellitic diimide (**5.1**–**5.4**, Figure 7).^[^ ^70^, ^71^ ^]^ Via π‐π stacking and the associated intermolecular CT, the original emission of DBSe in **5.2**, and **5.4** could be enhanced and bathochromically shifted toward the green region in the co‐crystalline arrangement. Whereas the co‐crystals with DBT and the diimide (**5.1**) exhibited a TADF and phosphorescence emission, the selenium‐containing congener (**5.2**) showed only phosphorescence, highlighting the enhanced triplet formation ability due to enhanced SOC. Additionally, the absence of TADF can be attributed to a higher energy gap between the S_1_ and T_1_ state, inhibiting rISC.^[^ ^70^ ^]^ This may be the reason for multiple applications of organoselenium and ‐tellurium compounds in optoelectronic devices.^[^ ^72^, ^73^ ^]^ The presence of a heavy chalcogen accelerates triplet formation,^[^ ^72^ ^]^ qualifying them for use in OLEDs or organic solar cells (OSCs).^[^ ^73^, ^74^ ^]^

Examining selenoethers, *Han* et al. synthesized the diaryl selenide **5.5**, showing emission in the near‐infrared region in oxidised form, leading to a redox‐responsive dye for bioimaging.^[^ ^75^ ^]^ Similar achievements were accomplished by *Kryman* et al. for rhodamine derivatives, revealing the tellurium congener **5.6** as an oxidation‐on‐off red emitter, applicable for visualising redox processes for bioimaging.^[^ ^75^ ^]^

*Rivard* et al. presented in 2015 for the first time a phosphorescent tellurophene doped by boronic esters (**5.7**, **5.8**, Figure 7).^[^ ^76^ ^]^ Thereby, the authors achieved green and orange emission colours only by varying the substitution pattern. In 2018, the authors replaced the borane groups with triphenylamine substituents (**5.9**, **5.10**), showing that the boronic esters are not required for phosphorescence in tellurophenes.^[^ ^77^ ^]^ By incorporating the tellurophene moiety into a phosphorescent macrocycle, a visually responsible host‐guest system could be achieved, whereby electron‐deficient aromatic compounds were incorporated as guests and resulted in quenched emission.^[^ ^78^ ^]^

In 2019, *Jiang* et al. presented a series of DBTe derivatives **5.11**‐**13**, scanning multiple substituents and substitution patterns in the resulting compounds in terms of photophysical properties.^[^ ^36^ ^]^ The authors highlighted three compounds showing RTP properties in solid state, whereas green (**5.11**), yellow (**5.12**), and orange (**5.13**) phosphorescence could be observed. Thereby, the latter compound showed multiple emission bands (468, 600 nm), which, the authors suggest, originate from excimer and monomer emission.^[^ ^36^ ^]^ By screening these simple compounds, it can be proposed that the 3‐position could be synthetically and photophysically promising for further functionalisation.

The shortage of luminescence studies on heavier DBCs is still not fully understood. Based on early investigations of the series of DBCs, their capability to exhibit triplet emission should qualify them for additional functionalities. While DBT is often used as a building block because of its triplet formation property, DBSe is capable of an even faster formation of triplet excitons,^[^ ^72^ ^]^ highlighting it as another promising building block for phosphorescent materials. However, DBSe has only been investigated via cocrystallization, leading to the question of why functionalized DBSe luminophores have not yet been reported. Perhaps one reason is the delicate balance between efficient ISC and inhibition of nonradiative decay. Due to the facilitated ISC processes, the population of the triplet manifold should be favored, leading to phosphorescence. Despite this, the enhanced SOC could also increase nonradiative decay of the triplet exciton as the transition from T_1_ to S_0_ is also dependent on the ISC rate. Consequently, the lifetime decreases with increasing atomic number due to easier ISC.

### 2.6 “Impurity” Doping

**Figure 8:**
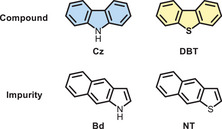
Structural formulas of Cz, DBT, and their isomers contained in commercial sources.

In 2021, *Liu* and colleagues disclosed that commercial carbazole contains an isomer of Cz that greatly enhances its phosphorescence.^[^ ^79^ ^]^ During the commercial refinement of Cz from anthracene oil, residual 1*H*‐benzo[*f*]indole (Bd) is not sufficiently removed (Figure 8). In contrast to commercial Cz with a *Φ*_PL_ of 70% and a visible afterglow of 2 s duration, Cz synthesized in the laboratory (Lab‐Cz) and carefully purified displayed 29% *Φ*_PL_ and no observable afterglow by the naked eye.^[^ ^80^ ^]^ Importantly, the authors were not able to sufficiently purify the Cz from TCI with their 4‐step purification process with repeated column chromatography and recrystallization. As a consequence, several literature‐known examples of Cz derivatives with reported long phosphorescence were revisited, and their properties were traced back to the impurity.^[^ ^79^ ^]^

Later on, the same phenomenon was uncovered for DBT: the RTP of commercial DBT could only be reproduced when highly pure Lab‐DBT was purposely doped with its structural isomer naphtho[2,3‐*b*]thiophene (NT, Figure 8).^[^ ^81^ ^]^ As an example, compound **1.2** (chapter 2.1) synthesized from Lab‐DBT showed noticeably weaker and shorter phosphorescence than previously reported (*Φ*_P_ = 0.06% and* τ*_P_ = 2.8 ms compared to 6.8% and 103.8 ms).^[^ ^49^, ^81^ ^]^

Since DBF is also derived from natural sources, the same problem may occur. Hence, in this field of research, it is critical to state whether the DBC was obtained commercially or by synthesis. Unless additional experiments were conducted, it can be neither confirmed nor excluded whether commercial impurities were sufficiently removed during multi‐step syntheses.

Furthermore, a recent review by *Mazarevics* et al. highlighted the generality of impurity‐induced RTP.^[^ ^82^ ^]^ They pointed out that it concerns plenty of organic compound classes, either by pre‐existing impurities or operational ones that emerge during synthesis. Since impurity contents of 0.1% or less can suffice to induce RTP, standard analytical methods like NMR or even HPLC may not be enough to exclude their involvement. Thus, the authors raised the question if any reported organic solid‐state phosphor was ever truly pure.

Aside from reproducibility problems that arise from impurity content variation between batches and different procedures, at worst, the underlying mechanisms for organic RTP may be falsely assigned. Hence, especially for the above‐mentioned compound classes, the results should be interpreted with caution. Nevertheless, in case impurities were at play in literature examples and the reasons were misattributed, the observed photophysical properties would exemplify for the potential of doping in organic phosphors.

## 3 Summary and Outlook

DBC are a class of versatile purely organic emitters which have been found to be capable of numerous applications from materials science to optoelectronics. In this contribution, we highlighted the state of the art in the synthesis and photophysics fine‐tuning of these fascinating compounds. General design concepts typically involve the introduction of carbonyl groups, sulfonyl groups, or amines to incorporate lone pairs and induce CT character, often in combination with heavy halogen atoms. Interestingly, only minor research has been conducted for the heavier congeners selenium and tellurium, which is peculiar, since heavier atoms often feature faster ISC and strong SOC. For sure, a delicate balancing between these features and emission, by preventing non‐radiative decay, is needed and will be a future challenge for scientists. Besides that, researchers around the world should be aware that minimal impurities, even in commercially available chemicals, may lead to a drastic manipulation of photophysical properties.

## Conflict of Interest

The authors declare no conflict of interest.

## Data Availability

Data sharing is not applicable to this article as no new data were created or analyzed in this study
